# Editorial: Recent advances in the mechanism and treatment of pituitary tumors

**DOI:** 10.3389/fneur.2023.1324189

**Published:** 2024-01-16

**Authors:** Wei Shi, Qianqian Liu, Feng Jia, Xuejian Wang

**Affiliations:** ^1^Department of Neurosurgery, Affiliated Hospital of Nantong University, Nantong University, Nantong, Jiangsu, China; ^2^Department of Neurosurgery, Renji Hospital, Shanghai, China; ^3^Department of Neurosurgery, Affiliated Hospital 2 of Nantong University, Nantong University, Nantong, Jiangsu, China

**Keywords:** recent advances, mechanisms, treatment, pituitary tumors, editorial

The understanding and continuous development of new technologies have propelled both basic and clinical research on pituitary tumors forward. Considering these advancements, we initiated the “*Recent advances in the mechanism and treatment of pituitary tumors*” Research Topic. In this Research Topic, we have included a total of seven articles. Our goal was to consolidate the latest technologies to advance pituitary tumor treatment and formulate enhanced treatment plans. In our consideration of articles, we leaned toward incorporating clinical studies, believing that the promotion of clinical treatment and technology holds greater significance and value in the current approach to pituitary tumor treatment, with surgery standing out as the primary intervention (1). While recent years have witnessed remarkable improvements in the surgical resection and cure rates of pituitary tumors due to the development of endoscopic technology, challenges such as cerebrospinal fluid leakage persist as major limiting factors. Addressing these concerns necessitates further discussion to optimize therapeutic outcomes (2–4).

The treatment of cerebrospinal fluid leakage and reconstruction of the skull base are pivotal techniques in endonasal pituitary tumor surgery. Different surgical units employ diverse approaches, necessitating thorough examination and effective communication of experiences. Presently, a variety of materials are available for skull base reconstruction, encompassing autologous, allogeneic, and artificial options (5, 6). Autologous materials consist of mucosal flaps, bone, fascia, and fat (7). Chen et al. proposed the utilization of an *in-situ* bone flap for skull base reconstruction, achieving a satisfactory therapeutic outcome. This technique offers the advantage of convenient material acquisition and a reliable source for reconstruction materials. Some experts advocate for the effectiveness of autologous fat reconstruction of the skull base (8). However, Wang et al. argue that intradural fat graft packing is unnecessary during endoscopic endonasal pituitary adenoma resection. This perspective aims to minimize additional trauma associated with fat harvesting.

The field of endoscopy technology has witnessed rapid development, gaining unanimous recognition, and progressing from traditional transnasal endoscopy to transcranial endoscopy (9). Despite the longstanding development of endonasal endoscopy, there are still variations in approaches, including single and double nostril techniques (10, 11). In terms of surgical approach technology, Cong et al. introduced the “endoscopic 112 transseptal approach,” which minimizes nasal mucosal injury. The use of this surgical approach not only avoids the operational inconvenience of single-nostril surgery, but also reduces the nostril injury of double-nostril surgery, which has its advantages and practical significance in clinical application. Additionally, Wu et al. proposed the “Endoscopic transcranial transdiaphragmatic approach” as an exemplary transcranioscopic method capable of tumor removal without inducing cerebrospinal fluid rhinorrhea. At present, the resection rate of large intrasellar and suprasellar tumors is not satisfactory. In this paper, by opening the sellar septum and combining with the use of “0” and “30” angle endoscopy, the tumor was more completely resected. But their limitations include its retrospective aspect and the fact that the cohort was relatively small to make definitive conclusions. This approach is inappropriate for giant pituitary adenomas (GPAs) with a shorter sella turcica length (distance from tuberculum sella to the tip of the dorsum sella). Besides, simultaneous mastering the manipulation of both the microscope and the endoscope requires extensive training and a long learning curve, because dissection of the giant tumors under an endoscopic view remains a great challenge for most neurosurgeons.

In the realm of tumor resection techniques, Zhang et al. proposed a method for treating pituitary tumors without relying on an intact pseudoenvelope. In recent years, there has been a growing comprehension of the false envelopes associated with pituitary tumors. This study delves into the characteristics of pituitary tumors lacking a complete pseudoenvelope and introduces treatment concepts and techniques. It represents a more profound exploration of existing pseudoenvelope technologies, holding substantial significance. Additionally, Nakaya et al. suggested that the volume of the sphenoid sinus could serve as a potential predictor of the extent of resection, enabling better visualization of pituitary neuroendocrine tumors (PitNETs) with cavernous sinus (CS) invasion. This study provides a novel examination of the correlation between paranasal sinus volume and tumor resection. The findings may contribute additional insights for future investigations.

In addition, we advocate for careful consideration of both typical and special cases during clinical treatment. For instance, Yan et al. illustrated a case of a thyrotropin-secreting pituitary macroadenoma with diffuse calcification. Conducting a literature review of special and rare cases is invaluable for fostering awareness and comprehension of such instances is important.

Fortunately, the continuous advancement and application of new technologies and concepts, particularly the ongoing progress in artificial intelligence technology (e.g., robots), are facilitating its integration into clinical practice. There remains ample opportunity for further advancement in the treatment of pituitary tumors. The promotion of clinical development hinges on the persistent exploration of novel technical approaches. Neurosurgeons are encouraged to actively engage in the development and implementation of emerging technologies crucial for elevating the cure rate of pituitary tumors.

## Author contributions

WS: Writing—original draft, Writing—review & editing. QL: Writing—original draft, Writing—review & editing. FJ: Writing—original draft, Writing—review & editing. XW: Writing—original draft, Writing—review & editing.
